# MicroRNA-217 functions as a prognosis predictor and inhibits colorectal cancer cell proliferation and invasion via an AEG-1 dependent mechanism

**DOI:** 10.1186/s12885-015-1438-z

**Published:** 2015-05-28

**Authors:** Bo Wang, Zhan-long Shen, Ke-wei Jiang, Gang Zhao, Chun-you Wang, Yi-chao Yan, Yang Yang, Ji-zhun Zhang, Chao Shen, Zhi-dong Gao, Ying-jiang Ye, Shan Wang

**Affiliations:** 1 Department of Gastroenterological Surgery, Peking University People’s Hospital, No.11 Xizhimen South Street, Xicheng District, Beijing, 100044 P.R. China; 2 Pancreatic Disease Institute, Union Hospital, Tongji Medical College, Huazhong University of Science and Technology, Wuhan, People’s Republic of China

**Keywords:** miR-217, AEG-1, colorectal cancer, proliferation, invasion

## Abstract

**Background:**

Recent studies have indicated the possible function of miR-217 in tumorigenesis. However, the roles of miR-217 in colorectal cancer (CRC) are still largely unknown.

**Methods:**

We examined the expression of miR-217 and AEG-1 in 50 CRC tissues and the corresponding noncancerous tissues by qRT-PCR. The clinical significance of miR-217 was analyzed. CRC cell lines with miR-217 upregulation and AEG-1 silencing were established and the effects on tumor growth *in vitro* and *in vivo* were assessed. Dual-luciferase reporter gene assays were also performed to investigate the interaction between miR-217 and AEG-1.

**Results:**

Our data demonstrated that miR-217 was significantly downregulated in 50 pairs of colorectal cancer tissues. MiR-217 expression levels were closely correlated with tumor differentiation. Moreover, decreased miR-217 expression was also associated with shorter overall survival of CRC patients. MiR-217 overexpression significantly inhibited proliferation, colony formation and invasiveness of CRC cells by promoting apoptosis and G0/G1 phase arrest. Interestingly, ectopic miR-217 expression decreased AEG-1 expression and repressed luciferase reporter activity associated with the AEG-1 3′-untranslated region (UTR). AEG-1 silencing resulted in similar biological behavior changes to those associated with miR-217 overexpression. Finally, in a nude mouse xenografted tumor model, miR-217 overexpression significantly suppressed CRC cell growth.

**Conclusions:**

Our findings suggest that miR-217 has considerable value as a prognostic marker and potential therapeutic target in CRC.

**Electronic supplementary material:**

The online version of this article (doi:10.1186/s12885-015-1438-z) contains supplementary material, which is available to authorized users.

## Background

Colorectal cancer (CRC) is the third most common cancer and the fourth most common cause of cancer deaths globally, accounting for approximately 1.2 million new cases and 600,000 deaths each year [1]. There is an urgent need to clarify the mechanisms underlying the pathogenesis of CRC and to develop novel and effective methods for its diagnosis and treatment [2, 3]. The identification of tissue-specific biomarkers with prognostic and therapeutic significance is, therefore, an important strategy [3, 4].

MicroRNAs (miRNAs) are small noncoding RNAs that induce degradation or translational repression of target gene mRNA. Recent evidence suggests that miRNAs are often aberrantly expressed in various cancers, and are correlated with prognosis and therapeutic outcomes in patients. In CRC, a number of miRNAs have been identified as regulators of cell proliferation and invasion, including miR-200a [5], miR-214 [6] and miR-221 [7]. Therefore, more extensive investigations are required to identify additional relevant miRNAs and to clarify the roles of these molecules in CRC.

MiR-217 has been reported to play an important role in carcinogenesis. In pancreatic cancer [8], hepatocellular carcinoma [9], renal cell carcinoma [10] and chronic myelogenous leukemia [11], miR-217 is downregulated and functions as a tumor suppressor, while it overexpressed and acts as an oncogene in B-cell lymphomas [12]. Moreover, miR-217 was demonstrated to modulate epithelia cell senescence in metabolic disorders [13]. However, the role of miR-217 in CRC remains to be elucidated. In 2009, Stuckenholz et al. [14] identified a number of novel genes, including miR-217, involved in mammalian gastrointestinal development, which were implicated as potential targets for therapeutic intervention in the management of gastrointestinal disease and cancer. Based on these findings, we hypothesized that miR-217 plays a role in human CRC. The present study compared the expression of miR-217 in CRC tissues and normal colorectal (CRN) tissues. Furthermore, the correlations between miR-217 and the clinical characteristics of CRC were analyzed.

Prediction software (TargetScan, microRNA and miRDB) analysis indicated that astrocyte-elevated gene-1 (AEG-1) is a potential target of miR-217. AEG-1, also known as metadherin (MTDH) or LYRIC, is induced in primary human fetal astrocytes infected with HIV-1 or treated with a recombinant HIV-1 envelope glycoprotein (gp120) [15]. AEG-1 has been reported to be significantly overexpressed and function as a key oncogenic factor in various tumors such as breast cancer [16], neuroblastoma [17], hepatocellular carcinoma [18], cervical cancer [19] and gastric cancer [20]. In CRC, ectopic expression of AEG-1 is observed in CRC tissues and high AEG-1 expression correlates with poor overall survival of patients [21, 22]. Furthermore, inhibition of AEG-1 expression resulted in suppression of proliferation and invasiveness of CRC cells, with modulation of MMP2 or AMPK signaling [23–25]. These findings suggest that AEG-1 promotes CRC. Therefore, in the current study, we investigated AEG-1 as the target for miR-217 to further explore the effects of miR-217/AEG-1 signaling on CRC.

## Methods

### Tissue samples and cell lines

Tissue samples were obtained from patients undergoing coloproctectomy according to the National Comprehensive Cancer Network (NCCN) guidelines for colon/rectal cancer (version 1. 2013). Samples were immediately snap-frozen and stored at −80 °C until RNA and protein extraction. All samples were identified as colorectal adenocarcinoma by two pathologists independently. All patients provided written informed consent before samples were collected and the study was approved by the local Research Ethics Committee of Peking University. The human CRC cell lines SW480, SW620, RKO, HT29, HCT116, and LoVo were purchased from the American Type Culture Collection (Manassas, VA, USA). NCM460 cells were purchased from INCELL Corporation (San Antonio, TX, USA). The genotypes of all cell lines were authenticated by DNA fingerprinting. All cells were cultured in RPMI1640 medium supplemented with 10 % fetal bovine serum (FBS) (all from Gibco), 100 IU/mL penicillin, and 100 μg/mL streptomycin at 37 °C under 5 % CO_2_.

### Quantitative real-time reverse transcription polymerase chain reaction (qRT-PCR)

Reverse transcription was performed with a reverse transcription kit (Takara, Japan). MiRNAs and potential target gene expression levels were measured by qRT-PCR with the SYBR Green PCR Kit (Takara) using the CFX96 Real-Time PCR Detection System (Bio-Rad, Hercules, CA, USA). Human U6 RNA or glyceraldehyde-3-phosphate dehydrogenase (GAPDH) RNA was amplified as an internal control. The RNA expression levels were calculated according to 2^-ΔΔCt^. MiR-217 expression was deemed to be high when the expression level was equal to or above the median of the cohort and low when it was below the median of the cohort [26]. Primer sequences are shown in Additional file 1: Table S1. The universal reverse primers provided by Takara were used for amplification of U6 and miR-217.

### Transfection

The miRNAs and siRNAs used in this study were designed and synthesized by RiboBio (Ribobio Co., Guangzhou, China). AEG-1 encoding plasmids were obtained from Invitrogen. Transfections with miRNA, siRNA or AEG-1 plasmids were performed using Lipofectamine 2000 (Invitrogen). CRC cells were seeded into 12-well plates before the transfection. The final concentration of miR-217 mimics or inhibitor or siRNA-AEG1 was 50 nM. The lentiviral miR-217 (LV-miR-217) and empty lentiviral (LV-miR-NC) vectors were generated by Genechem Company (Shanghai, China) and were used to transfect CRC cells according to the manufacturer’s instructions. All oligonucleotide sequences used in this experiment are listed in Additional file 1: Table S2.

### Western blot assays

CRC cells were collected at 48 h after treatment with 50 nM miR-217 mimics or inhibitor or siRNA-AEG1 and corresponding controls. Protein extraction, SDS-PAGE gel electrophoresis and blotting were performed as previously described [27]. Details of the primary detection antibodies are shown in Additional file 1: Table S3.

### Cell proliferation and colony formation assays

The CCK8 colorimetric assays (Dojindo, Kyushu, Japan) were performed to estimate the cell proliferation rate according to the manufacturer’s protocol. The cells were incubated for 4 h after adding the CCK8 reagents. Proliferation at different time-points was assessed by measuring the absorbance at 450 nm using a microplate reader (Bio-Rad). The CCK8 assay was repeated three times with six replicates.

For colony formation assays, transfected cells were seeded into 6-well plate, incubated for 10 days and then stained with 0.1 % crystal violet. The colony assay was repeated three times using duplicate samples.

### Evaluation of cell cycle distribution and apoptosis

These assays were performed by BD Biosciences flow cytometry as previously reported [28]. For cell cycle assays, cells were collected and stained using BD cycletest^TM^ plus DNA reagent kit (BD Biosciences) according to the manufacturer’s instructions. For cell apoptosis analysis, cells were collected 72 h after transfection, and the assays were performed with the Alexa Fluor^R^488 annexin V/Dead cell apoptosis kit (Invitrogen). Data were analyzed with FlowJo V7 software (Tree Star, Ashland, OR, USA).

### Invasion assay

Transwell assays were performed to evaluate the invasive ability of CRC cells. Briefly, cells were seeded in the upper chamber (24-well plates, 8-μm pore size, Corning) with media containing 0.1 % bovine serum albumin and media containing 30 % FBS was placed in the lower chamber. After culture for 48 h, invasive cells at the bottom of the membrane were stained with 0.1 % crystal violet and were counted under a microscopic. Invasion assays were repeated three times using duplicate samples.

### Dual-luciferase assay

MiR-217-binding region of AEG-1 was identified by TargetScan 6.2 (http://www.targetscan.org/). SW480 cells were seeded in 96-well plates and cotransfected with total of 100 ng pMIR-REPORT Luciferase vector (Ribobio Co.) containing the AEG-1 3′UTR (0–1,500 bp) or mutated sequences plus 50 nM miR-217 mimics or negative control (NC) mimic according to the manufacturer’s instructions. After incubation for 48 h, luciferase activity was determined using the dual-luciferase reporter assay system (Promega, Madison, WI, USA). The relative luciferase activities were determined by normalizing to Renilla Luciferase activities.

### CRC xenograft model

Four-week-old female BALB/c-nude mice (Vital River Laboratories, Beijing, China) were used to investigate SW480 cell tumorigenicity. A total of 200 μL cell suspensions (containing 1 × 10^7^ SW480 cells) were subcutaneously injected into the right flank of the mice. Tumor volumes were measured every 4 days and calculated according to V = 0.5 × L (length) × W^2^ (width). At 32 days after the cell inoculation, mice were sacrificed and tumors were excised to measure the volume. All animal experiments were reviewed and approved by the Animal Research Committee of the Peking University People’s Hospital. Care and handling of the animals was performed in accordance with the guidelines of the Institutional and Animal Care and Use Committees.

### Statistical analysis

Unless otherwise specified, all results were expressed as mean ± SD and analyzed using the SPSS 20.0 software (SPSS, Chicago, IL, USA). Differences between groups were assessed using Student’s *t*-test and Fisher’s exact test. The relationship between miR-217 expression and the clinicopathologic features of CRC was analyzed using the Pearson χ^2^ test. The differences between the two patient groups were analyzed by log-rank tests and the Kaplan–Meier method was used to calculate the overall survival. *P* < 0.05 was considered to indicate statistical significance.

## Results

### Clinicopathologic significance of miR-217 in CRC patients

**Fig. 1:**
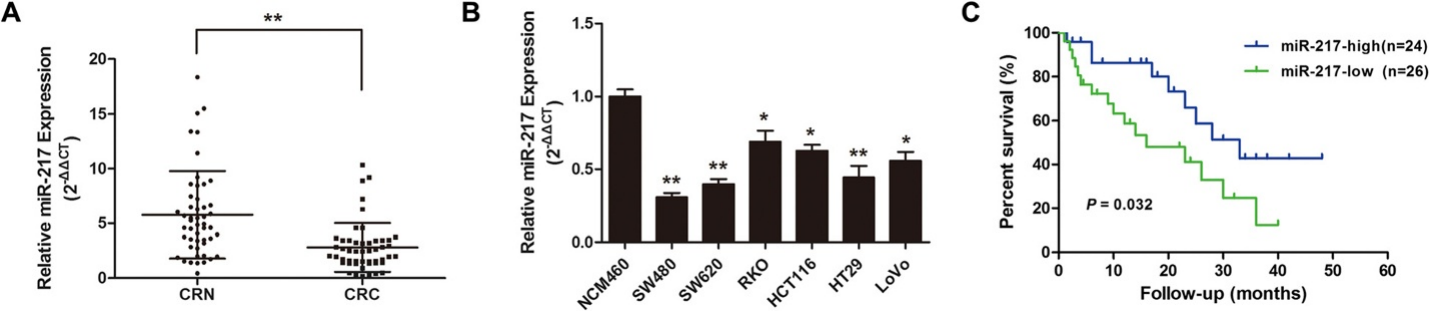
Determining miR-217 expression in CRC tissues and cell lines and its clinical significance. (**a**) Relative expression level of miR-217 in human CRC tissues (n = 50) and CRN tissues (n = 50), examined by qRT-PCR. CRC: colorectal cancer tissues; CRN: matched adjacent noncancerous colorectal tissues. (**b**) The relative miR-217 expression level in six CRC cell lines compared with the normal colorectal cell line NCM460. The average gene expression from NCM460 was appointed as 1. (**c**) Kaplan-Meier survival curve for CRC patients with miR-217-high (n = 24) and miR-217-low (n = 26) character. P value was obtained by a log-rank test. *P < 0.05, **P < 0.01

qRT-PCR analysis showed that miR-217 expression was significantly decreased in CRC tissue samples compared with the corresponding CRN tissue samples (Fig. 1a). Furthermore, all six CRC cell lines expressed lower levels of miR-217 than the normal colorectal cell line NCM460 (Fig. 1B). The SW480 and SW620 cell lines exhibited the lowest levels of miR-217 expression and were therefore, selected for use in subsequent studies.

The association of miR-217 expression with CRC prognosis was also investigated. The analysis of clinical pathological characteristics showed that low miR-217 expression was significantly associated with poor tumor differentiation (*P* = 0.038), but not with patient age, gender, tumor size, TNM stage, lymph node metastasis, or distant metastasis and vessel infiltration in CRC (Table 1). Moreover, Kaplan–Meier analysis revealed that CRC patients with low miR-217 expression had a significantly shorter median survival (19.5 ± 2.9 vs. 32.0 ± 3.8 months, *P* = 0.032; Fig. 1C) than those with high miR-217 expression. Furthermore, Cox’s multivariate analysis showed that miR-217 expression, TNM stage and distant metastasis were significantly related to overall survival of CRC patients as independent prognostic factors (Table 2). These results demonstrate that lower miR-217 expression levels indicate poorer prognosis in CRC patients.

**Table 1:** The relationship between miR-217 expression and clinicopathologic characteristics in CRC patients

miR-217 expression				
Parameters	High (n = 24)	Low (n = 26)	Total (n = 50)	*P* value
Age (y)				
≤60	9	11	20	0.729
>60	15	15	30	
Gender				
Female	7	14	21	0.077
Male	17	12	29	
Tumor size (cm)				
≤2	11	9	20	0.412
>2	13	17	30	
Tumor differentiation				
Well/moderate	18	12	30	0.038*
Poor	6	14	20	
TNM stage				
I + II	12	12	24	0.786
III + IV	12	14	26	
Lymph node metastasis				
Positive	11	13	24	0.768
Negative	13	13	26	
Distant metastasis				
Positive	7	7	14	0.860
Negative	17	19	36	
Vascular infiltration				
Positive	10	12	22	0.749
Negative	14	14	28	

*Statistically significant (P < 0.05)

**Table 2:** Multivariate analysis of factors associated with overall survival in CRC patients

	Multivariate analysis	
Variable	HR (95 % CI)	*P* value
Age	1.001 (0.966-1.038)	0.936
Gender	1.296 (0.448-3.749)	0.632
Tumor size	1.583 (0.569-4.404)	0.379
Tumor differentiation	1.646 (0.578-4.683)	0.350
TNM stage	0.132 (0.028-0.623)	0.010*
Lymph node metastasis	0.901 (0.355-2.292)	0.828
Distant metastasis	13.508 (2.770-65.864)	0.001*
miR-217	0.312 (0.111-0.877)	0.027*
AEG-1	1.228 (0.419-3.594)	0.708

HR, hazard ratio; CI, confidence interval

*Statistically significant (P < 0.05)

### MiR-217 repressed proliferation of CRC cell lines *in vitro* and *in vivo*

**Fig. 2:**
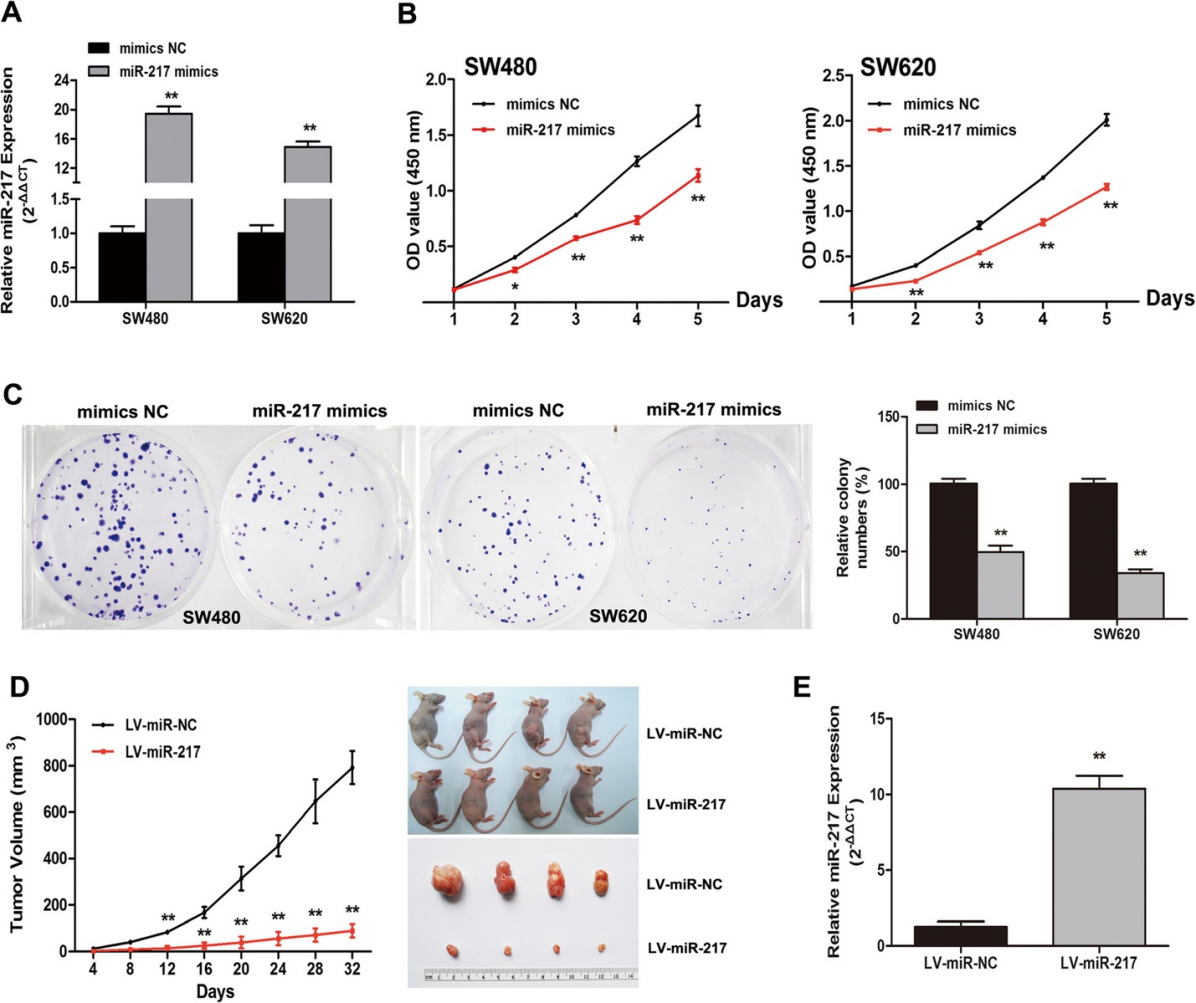
MiR-217 inhibits the growth of CRC cell lines *in vitro* and *in vivo*. (**a**) The relative expression level of miR-217 when transfected with miR-217 mimics and mimics NC measured by qRT-PCR. The average miRNA expression from mimics NC was appointed as 1. (**b**) The proliferation curve of SW480 and SW620 cells after transfected with miR-217 mimics by CCK8 assay. (**c**) Assessment of colony formation when upregulation of miR-217 expression. (**d**) The effects of overexpression of miR-217 on xenograft tumor growth in mice. Tumor growth curves were drew by measuring the subcutaneous tumor volumes every 4 days. Data are presented as mean ± SD. (**e**) Relative miR-217 expression level in excised tumors. *P < 0.05, **P < 0.01

Because miR-217 was expressed at low levels in the CRC cell lines, miR-217 gain-of-function studies were conducted using a strategy of transient transfection with miR-217 mimics. As shown in Fig. 2a, after transfection with miR-217 mimics, a 19.46-fold and 14.89-fold increase in miR-217 expression was observed in SW480 and SW620 cells, respectively. CCK8 assay showed that the proliferation rate of SW480 and SW620 cells were both significantly repressed after transfection with miR-217 mimics (Fig. 2b). Moreover, the colonies formed by cells transfected with miR-217 mimics were obviously fewer in number and smaller in size than those formed by the control cells (Fig. 2c).

These findings were confirmed in a CRC xenograft model *in vivo*. Xenografted tumors in mice inoculated with LV-miR-217-infected SW480 cells grew much more slowly than those in mice inoculated with the LV-miR-NC (Fig. 2d). qRT-PCR analysis showed that miR-217 expression levels were obviously increased in LV-miR-217-infected tumors compared with those in the control tumors (Fig. 2e). These results indicate that miR-217 suppressed proliferation of CRC cell lines both *in vitro* and *in vivo*.

### MiR-217 induced apoptosis and led to cell cycle arrest in CRC cell lines

**Fig. 3:**
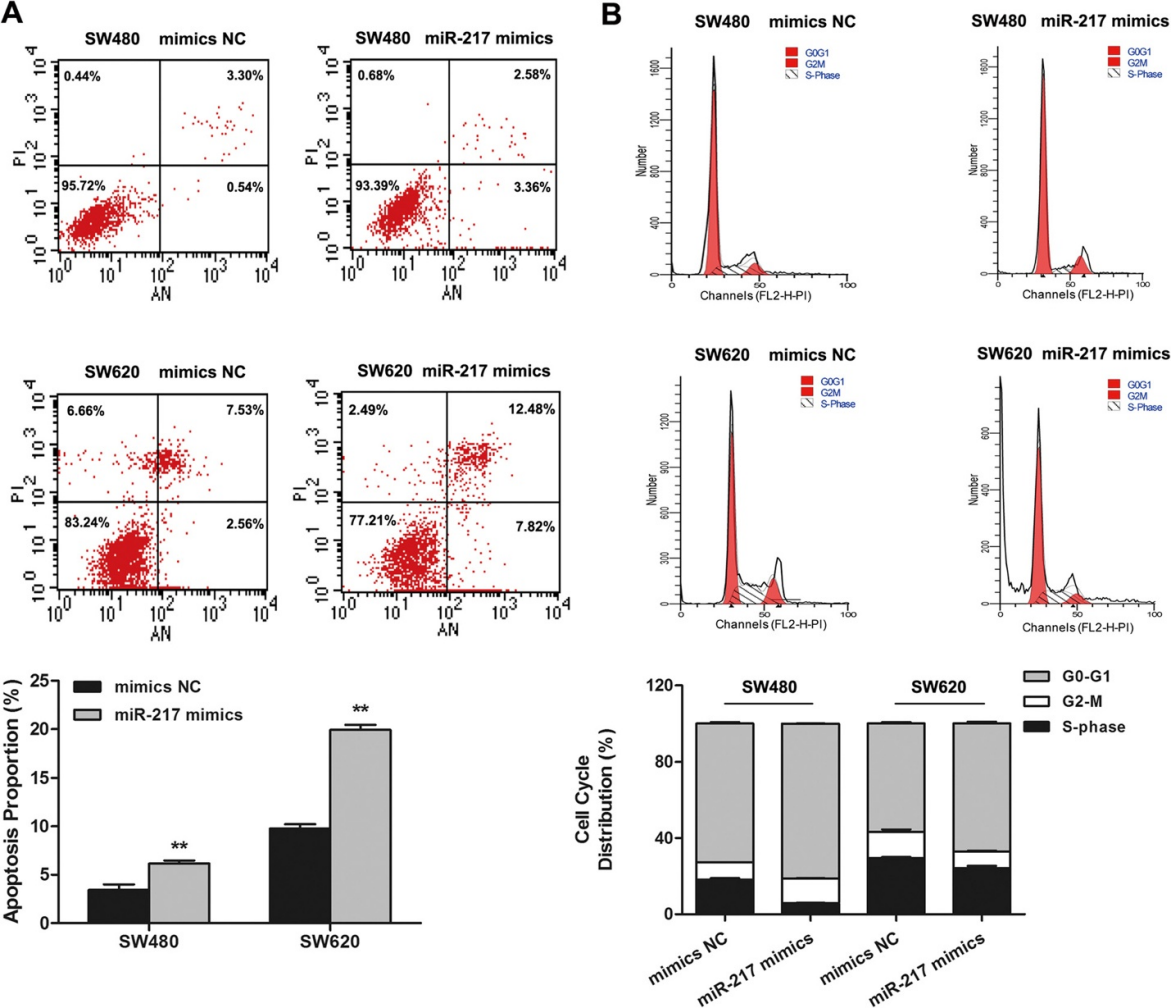
Overexpression of miR-217 enhances apoptosis and promotes G0/G1 phase arrest in CRC cells. (**a**) Flow cytometry analysis showed that after transfection with miR-217 mimics and mimics NC, the apoptosis rates of both SW480 and SW620 cells were markedly increased. (**b**) Cell cycle distribution assay was also applied using flow cytometry and treated with mimics as mentioned above. The histogram showed that miR-217 induced cell cycle arrest at G0/G1 phase. **P < 0.01

To elucidate the mechanism by which miR-217 expression affects cell proliferation, flow cytometry was employed to analyze the effects of miR-217 overexpression on CRC cell line apoptosis and cell cycle progression. As shown in Fig. 3a, the total apoptosis rate in cells transfected with miR-217 mimics was significantly increased compared with that in cells transfected with NC mimics (SW480, 6.16 ± 0.31 % vs. 3.44 ± 0.57 %, *P* < 0.01; SW620, 19.93 ± 0.52 % vs. 9.77 ± 0.45 %, *P* < 0.01). Moreover, for cell cycle analysis, the proportion of miR-217 mimic-transfected cells in the G0/G1 phase increased compared with that of the controls (SW480, 81.16 ± 0.06 % vs. 72.78 ± 0.71 %, *P* < 0.01; SW620, 66.94 ± 0.91 % vs. 56.69 ± 0.70 %, *P* < 0.01; Fig. 3B). These results demonstrate that ectopic expression of miR-217 promotes apoptosis and G0/G1 phase arrest.

### MiR-217 suppressed the CRC cell invasive activity

**Fig. 4:**
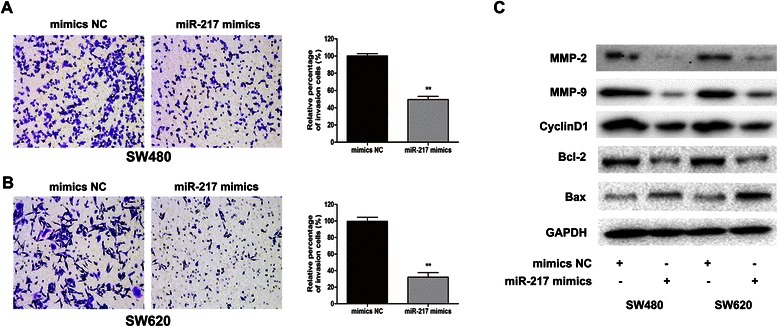
Ectopic miR-217 expression inhibits invasion of SW480 and SW620 cells. (**a**) Transwell method was applied to assess SW480 cells’ invasion ability after transfected with miR-217 mimics and negative control. (**b**) The invasiveness of SW620 cells was evaluated as mentioned above. Statistics analysis was performed by counting the stained cells that invaded to the lower chamber. All measurements were repeated three times in duplicate. (**c**) Western blot analysis showed the expression levels of invasion related molecules MMP2 and MMP9, cell cycle related protein cyclinD1, and apoptosis associated protein Bax and Bcl-2 after overexpression of miR-217.**P < 0.01

The effect of miR-217 on cell invasive capability was investigated in transwell experiments. The numbers of invading cells on the underside of the membrane were significantly reduced both in SW480 cells (Fig. 4a, P < 0.01) and SW620 cells (Fig. 4B, *P* < 0.01) when transfected with miR-217 mimics compared to those transfected with NC mimics. These results imply that miR-217 participates in the regulation of the CRC cell invasiveness.

### MiR-217 was involved in changes in the expression of molecules associated with invasion, cell cycle and apoptosis

The expression of the related proteins, MMP-2, MMP-9, cyclinD1 and Bcl-2 was significantly downregulated in cells transfected with miR-217 mimics, whereas the expression of Bax was increased in both SW480 and SW620 cells (Fig. 4c).

### MiR-217 suppressed AEG-1 expression by binding to its 3′UTR sequence

**Fig. 5:**
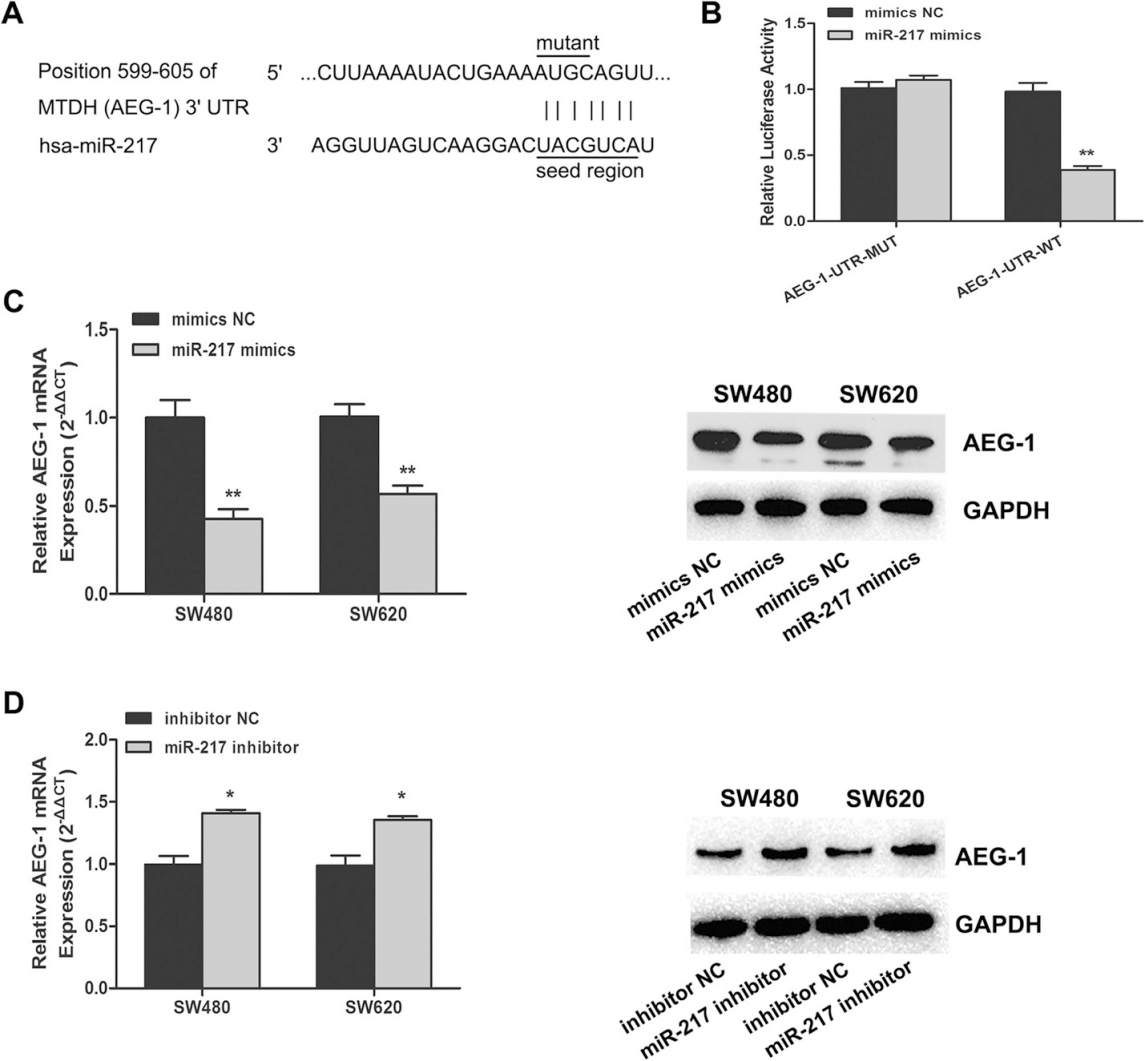
MiR-217 directly targets AEG-1 in SW480 cells. (**a**) Predicted binding site of human miR-217 to the 3’UTR of AEG-1 by TargetScan. (Top panel) The mutation of miR-217 binding site in the 3’UTR of AEG-1. (**b**) The reporter plasmids containing wild-type or mutant 3’UTR of AEG-1 was co-transfected with miR-217 mimics or mimics NC. The assay was performed twice in triplicate. The relative luciferase activity was obtained by Firefly luciferase activity normalized against Renilla luciferase activity. (**c**) The effects of overexpression of miR-217 on AEG-1 expression at mRNA level and protein level. (**d**) The effects of inhibition of miR-217 on AEG-1 expression at mRNA level and protein level. *P < 0.05, **P < 0.01

To clarify the underlying molecular mechanism of the suppressive effects of miR-217 on the proliferation and invasive capacity of CRC cells, we used bioinformatics methods (TargetScan, microRNA and miRDB) to search for potential target genes of miR-217. All prediction analyses indicated that AEG-1 is a potential target of miR-217. We then cloned the 3′UTR of AEG-1 containing wild-type or mutant seed-sequence-recognizing sites into a dual-luciferase reporter (Fig. 5a). After cotransfection of SW480 cells with miR-217 mimics or NC mimics and AEG-1-UTR-WT or AEG-1-UTR-MUT plasmids, luciferase activity was analyzed. Our results showed that miR-217 significantly decreased the relative luciferase activity in the reporter containing the wild-type 3′UTR, whereas the luciferase activity of the mutant was unaffected (Fig. 5b). We further evaluated the expression levels of AEG-1 mRNA and protein after modulation of miR-217 expression. As shown in Fig. 5c, transfection of CRC cells with miR-217 mimics led to a remarkable downregulation in AEG-1 mRNA and protein levels. In contrast, treatment with the miR-217 inhibitor caused an increase in AEG-1 mRNA and protein expression (Fig. 5d). These data indicate that miR-217 directly targets its predicted AEG-1 seed region.

### The correlation between miR-217 and AEG-1 expression in colorectal tissue samples

**Fig. 6:**
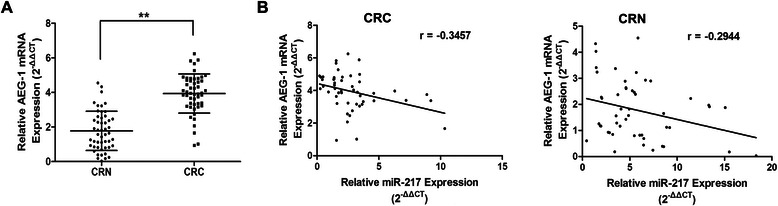
AEG-1 is upregulated in CRC tissues and negatively correlated with the expression level of miR-217 in both CRC and CRN tissue samples. (**a**) Upregulation of AEG-1 was observed in CRC tissue samples compared with that in adjacent CRN ones by qRT-PCR. (**b**) The analysis (Pearson’s correlation) of relationship between expression levels of AEG-1 and miR-217 in CRC and CRN tissues, respectively. **P < 0.01

We further analyzed the relationship between miR-217 and AEG-1 expression in colorectal tissue samples. First, we measured the AEG-1 mRNA levels in CRC tissues and the corresponding adjacent normal tissues by qRT-PCR. As shown in Fig. 6a, AEG-1 expression was much higher in CRC tissues than in the corresponding CRN tissues (*P* < 0.01). Interestingly, Pearson correlation analysis revealed an obvious inverse correlation was observed between miR-217 and AEG-1 expression both in CRC tissues (r = −0.3457, *P* < 0.05) and in CRN tissues (r = −0.2944, *P* < 0.05) (Fig. 6b).

### The effect of AEG-1 expression on the survival of CRC patients

Kaplan–Meier analysis indicated that there were no significant differences in the median survival of CRC patients with either low or high AEG-1 expression (Additional file 2: Figure S1).

### Silencing of AEG-1 inhibits malignant behavior of colorectal cancer cells

To further investigate the role of AEG-1 in CRC cells, AEG-1 siRNA was used to knockdown AEG-1 expression. AEG-1 expression was greatly decreased at both the mRNA and protein levels (Additional file 3: Figure S2A) after transfection with siRNA-AEG1. Similar to overexpression of miR-217, AEG-1 silencing markedly suppressed cell proliferation, colony formation, and invasive capacity, while G0/G1 arrest, and apoptosis were promoted (Additional file 3: Figure S2b-f).

### Restoration of AEG-1 expression contributed to the reversal of malignant behavior in SW620 cells

To confirm the role of AEG-1 in the anti-cancer effects of miR-217 in CRC cells, we restored AEG-1 expression by AEG-1 plasmid transfection after transfection with miR-217 mimics. As shown in Additional file 4: Figure S3, overexpression of both miR-217 and AEG-1 in SW620 cells caused no significant effects on cell proliferation, invasive capacity, cell cycle and apoptosis.

## Discussion

MiRNAs are known to be play a key role in tumorigenesis as a result of their involvement in many cellular processes including cell proliferation, differentiation, apoptosis and invasion [29, 30]. In the present study, we focused on miR-217, which is abnormally expressed in a variety of cancer types [8–10, 12]. To date, the evidence for aberrant expression of miR-217 in CRC has been obtained in microarray studies. In our study, qRT-PCR analysis demonstrated that miR-217 was significantly downregulated in CRC tissue samples and cancer cell lines. Furthermore, our study revealed, for the first time, the involvement of miR-217 in tumorigenesis through targeting AEG-1 targeting and that decreased miR-217 expression correlated with poor prognosis in patients with CRC. These findings implicate miR-217 as a novel prognostic marker in CRC.

Moreover, analysis of clinical data indicated that reduced expression of miR-217 in CRC patients correlated with poor tumor differentiation. In addition, Cox’s multivariate analysis indicated that miR-217 expression, TNM stage and distant metastasis act as an independent factor in the prediction of overall survival among patients with CRC.

In this study, we also showed, for the first time, that overexpression of miR-217 significantly repressed CRC cell proliferation, colony formation, and induced G0/G1 cell cycle arrest and apoptosis. Moreover, our *in vivo* studies confirmed that miR-217 overexpression remarkably suppressed CRC xenograft tumor growth in nude mice. These results imply that miR-217 acts as an inhibitor of colorectal tumorigenesis.

Metastasis, one of the most critical hallmarks of cancer, is the leading cause of cancer-related deaths worldwide, particular in CRC [31, 32]. Accumulating evidence demonstrates the close correlation of invasive capacity and metastasis with miRNAs, such as miR-124 in nasopharyngeal carcinoma [33], miR-153 in CRC [34] and miR-335 in lung cancer [35]. This evidence elucidating the role of miRNAs in CRC metastasis might represent the basis of a new therapeutic approach for CRC. The clinical outcomes in the patients in this study revealed that the expression level of miR-217 was closely correlated with CRC distant metastasis and also acted as an independent prognostic factor in patients with CRC. It is well-known that invasive tumors exist within a complex microenvironment composed of extracellular matrix (ECM) proteins, which play important roles in tumor invasion and metastasis [36]. Thus, matrigel invasion assays were performed in our study to mimic this environment. The results showed that after overexpression of miR-217, the invasion capability of CRC cells was significantly reduced, indicating the involvement of miR-217 in CRC invasion and metastasis. Thus, it can be hypothesized that restoration of miR-217 in CRC might be a new therapeutic approach in CRC, especially in CRC with distant metastasis.

The effects of miRNAs are largely dependent on their regulation of the expression of many cancer-related genes through post-transcriptional repression [37]. Using bioinformatics analysis, we found that miR-217 targeted multiple cancer-related genes that have been reported to have a close link with cancers, such as KRAS (pancreatic cancer) [8], E2F3 (hepatocellular carcinoma) [9], and DACH1 (breast cancer) [38]. Interestingly, in this study, AEG-1 was predicted to be one of the target genes of miR-217. AEG-1 expression is frequently increased in multiple cancers including CRC [21–23] and plays a critical role in oncogenic transformation and angiogenesis, which are essential to tumor cell development, growth, and metastatic progression [39–41]. These studies provide important insights and a unique perspective on this multifunctional oncogene. In the current study, we evaluated the prognostic value of AEG-1 in CRC patients. Although the survival analysis showed no significant difference between the AEG-1-low and AEG-1-high groups, the median survival time was longer in the patients with low AEG-1 expression. Moreover, knockdown of AEG-1 repressed cell growth and invasion, induced G0/G1 arrest and apoptosis, which was similar to the effects of miR-217 overexpression. Thus, the results of our study indicate that AEG-1 acts as a tumor promoter in CRC.

We next used dual-luciferase assays to determine whether miR-217 binds directly to the 3′UTR of AEG-1 mRNA. Ectopic expression of miR-217 resulted in significant AEG-1 downregulation at both the mRNA and protein levels, whereas miR-217 silencing led to restoration of AEG-1 expression. Furthermore, the expression level of AEG-1 was inversely correlated with the miR-217 expression in both CRC and CRN tissues. Therefore, the results indicate that decreased AEG-1 expression represents a mechanism by which miR-217 plays a role in the progression of cancer. To further clarify this point, we performed a rescue experiment which demonstrated that AEG-1 overexpression significantly reversed miR-217-induced apoptosis, cell cycle arrest, proliferative inhibition and invasive suppression of SW620 cells. However, the subcutaneous xenograft model in our study cannot sufficiently represent clinical CRC, especially with regard to metastasis [42]. The present study demonstrates that miR-217 remarkably represses the invasive ability of CRC cells *in vitro*; therefore, further investigations in a metastasis model are required to clarify the effects of miR-217 on invasion and metastasis of CRC *in vivo*.

## Conclusions

In this study we show that miR-217 is significantly downregulated in CRC and that decreased miR-217 expression levels indicate poor prognosis of CRC patients. In addition, our results indicate that miR-217 may suppress the tumorigenesis and aggressiveness of CRC through directly targeting AEG-1. Importantly, our findings implicate miR-217 as a prognostic marker and potential target for miRNA-based CRC therapy.

## Additional files

Additional file 1: Table S1. Primer sequences of genes. Table S2. Oligonucleotide sequences for transfection. Table S3. Western Blot primary antibodies.
Additional file 2: Figure S1. The effect of AEG-1 expression level on survival of CRC patients. Kaplan-Meier survival curve for CRC patients with AEG-1-high (n = 26) and AEG-1-low (n = 24) character. P value was obtained by a log-rank test.
Additional file 3: Figure S2. Knockdown of AEG-1 inhibit malignant biological behavior in SW480 and SW620 cell lines. (A) AEG-1 expression was downregulated after treated with siRNA-AEG-1 determined by qRT-PCR (left) and Western blot analysis (right). (B) Inhibition of AEG-1 expression repressed cell proliferation of SW480 and SW620 cells. (C) Silencing of AEG-1 led to repression of colony formation. (D) Knockdown of MAP4K4 weakened the ability of cell invasion. (E) Cell cycle was examined by flow cytometry. Silencing of MAP4K4 in SW480 and SW620 cells led to G0/G1 arrest. (F) The percentage of apoptotic cells increased through downregulation of AEG-1 in SW480 and SW620 cell lines. *P < 0.05, **P < 0.01.
Additional file 4: Figure S3. Rescue of miR-217 ectopic expression effects by simultaneous overexpression of AEG-1. (A) Cell proliferation detected in SW620 cells at 1, 2, 3, 4 and 5 days after transfection. (B) Results of SW620 cell invasion across an 8-μm pore size membrane with Matrigel. (C) Cell cycle determined in SW620 cells 48 h after transfection by Propidium-iodide staining flow cytometry. (D) Cell apoptosis detected by Annexin-V/propidium iodide combined labeling flow cytometry in SW620 cells 48 h after transfection. *P < 0.05, **P < 0.01.

## Abbreviations

CRC: colorectal cancer
CRN: colorectal normal
qRT-PCR: quantitative real-time reverse transcription polymerase chain reaction
UTR: untranslated region
GAPDH: glyceraldehyde-3-phosphate dehydrogenase
CCK8: cell counting kit-8
NC: negative control

## References

[1] Ferlay J, Shin HR, Bray F, Forman D, Mathers C, Parkin DM (2010). Estimates of worldwide burden of cancer in 2008: GLOBOCAN 2008. Int J Cancer.

[2] Akagi Y, Kinugasa T, Adachi Y, Shirouzu K (2013). Prognostic significance of isolated tumor cells in patients with colorectal cancer in recent 10-year studies. Mol Clin Oncol.

[3] Guo Y, Xu F, Lu T, Duan Z, Zhang Z (2012). Interleukin-6 signaling pathway in targeted therapy for cancer. Cancer Treat Rev.

[4] Jemal A, Bray F, Center MM, Ferlay J, Ward E, Forman D (2011). Global cancer statistics. CA Cancer J Clin.

[5] Pichler M, Ress AL, Winter E, Stiegelbauer V, Karbiener M, Schwarzenbacher D (2014). MiR-200a regulates epithelial to mesenchymal transition-related gene expression and determines prognosis in colorectal cancer patients. Br J Cancer.

[6] Chen DL, Wang ZQ, Zeng ZL, Wu WJ, Zhang DS, Luo HY (2014). Identification of MicroRNA-214 as a negative regulator of colorectal cancer liver metastasis by way of regulation of fibroblast growth factor receptor 1 expression. Hepatology.

[7] Liu S, Sun X, Wang M, Hou Y, Zhan Y, Jiang Y, Liu Z, Cao X, Chen P, Liu Z et al.: A microRNA 221- and 222-Mediated Feedback Loop, via PDLIM2, Maintains Constitutive Activation of NFkappaB and STAT3 in Colorectal Cancer Cells. Gastroenterology 2014 . 10.1053/j.gastro.2014.06.006 PMC4839969 24931456

[8] Zhao WG, Yu SN, Lu ZH, Ma YH, Gu YM, Chen J (2010). The miR-217 microRNA functions as a potential tumor suppressor in pancreatic ductal adenocarcinoma by targeting KRAS. Carcinogenesis.

[9] Su J, Wang Q, Liu Y (2014). miR-217 inhibits invasion of hepatocellular carcinoma cells through direct suppression of E2F3. Mol Cell Biochem.

[10] Li H, Zhao J, Zhang JW, Huang QY, Huang JZ, Chi LS (2013). MicroRNA-217, down-regulated in clear cell renal cell carcinoma and associated with lower survival, suppresses cell proliferation and migration. Neoplasma.

[11] Nishioka C, Ikezoe T, Yang J, Nobumoto A, Tsuda M, Yokoyama A (2014). Downregulation of miR-217 correlates with resistance of Ph(+) leukemia cells to ABL tyrosine kinase inhibitors. Cancer Sci.

[12] de Yebenes VG, Bartolome-Izquierdo N, Nogales-Cadenas R, Perez-Duran P, Mur SM (2014). miR-217 is an oncogene that enhances the germinal center reaction. Blood.

[13] MicroRNA 217 Modulates Endothelial Cell Senescence via Silent Information Regulator 1. Circulation 2009 :1524–1532. 10.1161/CIRCULATIONAHA.109.864629 19786632

[14] Stuckenholz C, Lu L, Thakur P, Kaminski N, Bahary N (2009). FACS-assisted microarray profiling implicates novel genes and pathways in zebrafish gastrointestinal tract development. Gastroenterology.

[15] Su ZZ, Kang DC, Chen Y, Pekarskaya O, Chao W, Volsky DJ (2002). Identification and cloning of human astrocyte genes displaying elevated expression after infection with HIV-1 or exposure to HIV-1 envelope glycoprotein by rapid subtraction hybridization. RaSH Oncogene.

[16] Li J, Yang L, Song L, Xiong H, Wang L, Yan X (2009). Astrocyte elevated gene-1 is a proliferation promoter in breast cancer via suppressing transcriptional factor FOXO1. Oncogene.

[17] Lee SG, Jeon HY, Su ZZ, Richards JE, Vozhilla N, Sarkar D (2009). Astrocyte elevated gene-1 contributes to the pathogenesis of neuroblastoma. Oncogene.

[18] He XX, Chang Y, Meng FY, Wang MY, Xie QH, Tang F (2012). MicroRNA-375 targets AEG-1 in hepatocellular carcinoma and suppresses liver cancer cell growth in vitro and in vivo. Oncogene.

[19] Liu X, Wang D, Liu H, Feng Y, Zhu T, Zhang L (2014). Knockdown of astrocyte elevated gene-1 (AEG-1) in cervical cancer cells decreases their invasiveness, epithelial to mesenchymal transition, and chemoresistance. Cell Cycle.

[20] Li G, Wang Z, Ye J, Zhang X, Wu H, Peng J, Song W, Chen C, Cai S, He YL et al.: Uncontrolled inflammation induced by AEG-1 promotes gastric cancer and is associated with poor prognosis. Cancer Res 2014 . 10.1158/0008-5472.CAN-14-0968 25092897

[21] Gnosa S, Shen YM, Wang CJ, Zhang H, Stratmann J, Arbman G (2012). Expression of AEG-1 mRNA and protein in colorectal cancer patients and colon cancer cell lines. J Transl Med.

[22] Song H, Li C, Li R, Geng J (2010). Prognostic significance of AEG-1 expression in colorectal carcinoma. Int J Colorectal Dis.

[23] Huang S, Wu B, Li D, Zhou W, Deng G, Zhang K (2014). Knockdown of astrocyte elevated gene-1 inhibits tumor growth and modifies microRNAs expression profiles in human colorectal cancer cells. Biochem Biophys Res Commun.

[24] Song H, Tian Z, Qin Y, Yao G, Fu S, Geng J (2014). Astrocyte elevated gene-1 activates MMP9 to increase invasiveness of colorectal cancer. Tumour Biol.

[25] Song HT, Qin Y, Yao GD, Tian ZN, Fu SB, Geng JS (2014). Astrocyte elevated gene-1 mediates glycolysis and tumorigenesis in colorectal carcinoma cells via AMPK signaling. Mediators Inflamm.

[26] Hwang JH, Voortman J, Giovannetti E, Steinberg SM, Leon LG, Kim YT (2010). Identification of microRNA-21 as a biomarker for chemoresistance and clinical outcome following adjuvant therapy in resectable pancreatic cancer. PLoS One.

[27] Zhao G, Wang B, Liu Y, Zhang JG, Deng SC, Qin Q (2013). MiRNA-141, downregulated in pancreatic cancer, inhibits cell proliferation and invasion by directly targeting MAP4K4. Mol Cancer Ther.

[28] Zhao G, Zhang JG, Liu Y, Qin Q, Wang B, Tian K (2013). miR-148b functions as a tumor suppressor in pancreatic cancer by targeting AMPKalpha1. Mol Cancer Ther.

[29] Bartel DP (2004). MicroRNAs: genomics, biogenesis, mechanism, and function. Cell.

[30] Winter J, Jung S, Keller S, Gregory RI, Diederichs S (2009). Many roads to maturity: microRNA biogenesis pathways and their regulation. Nat Cell Biol.

[31] De Roock W, De Vriendt V, Normanno N, Ciardiello F, Tejpar S (2011). Mutations: implications for targeted therapies in metastatic colorectal cancer. Lancet Oncol.

[32] Carpizo DR, D'Angelica M (2009). Liver resection for metastatic colorectal cancer in the presence of extrahepatic disease. Lancet Oncol.

[33] Peng XH, Huang HR, Lu J, Liu X, Zhao FP, Zhang B (2014). MiR-124 suppresses tumor growth and metastasis by targeting Foxq1 in nasopharyngeal carcinoma. Mol Cancer.

[34] Zhang L, Pickard K, Jenei V, Bullock MD, Bruce A, Mitter R (2013). miR-153 supports colorectal cancer progression via pleiotropic effects that enhance invasion and chemotherapeutic resistance. Cancer Res.

[35] Gong M, Ma J, Guillemette R, Zhou M, Yang Y, Yang Y (2014). miR-335 inhibits small cell lung cancer bone metastases via IGF-IR and RANKL pathways. Mol Cancer Res.

[36] Leeman MF, Curran S, Murray GI (2003). New insights into the roles of matrix metalloproteinases in colorectal cancer development and progression. J Pathol.

[37] Peter ME (2010). Targeting of mRNAs by multiple miRNAs: the next step. Oncogene.

[38] Zhang Q, Yuan Y, Cui J, Xiao T, Jiang D (2015). MiR-217 Promotes tumor proliferation in breast cancer via targeting DACH1. J Cancer Educ.

[39] Wang YP, Liu IJ, Chiang CP, Wu HC (2013). Astrocyte elevated gene-1 is associated with metastasis in head and neck squamous cell carcinoma through p65 phosphorylation and upregulation of MMP1. Mol Cancer.

[40] Kochanek DM, Wells DG (2013). CPEB1 regulates the expression of MTDH/AEG-1 and glioblastoma cell migration. Mol Cancer Res.

[41] Srivastava J, Siddiq A, Emdad L, Santhekadur PK, Chen D, Gredler R (2012). Astrocyte elevated gene-1 promotes hepatocarcinogenesis: novel insights from a mouse model. Hepatology.

[42] Fu X, Guadagni F, Hoffman RM (1992). A metastatic nude-mouse model of human pancreatic cancer constructed orthotopically with histologically intact patient specimens. Proc Natl Acad Sci U S A.

