# Supplementary material for: MicroRNA-217 functions as a prognosis predictor and inhibits colorectal cancer cell proliferation and invasion via an AEG-1 dependent mechanism
Source: BMC Cancer. 2015 May 28;15:437. doi: 10.1186/s12885-015-1438-z (PMC4446846; doi:10.1186/s12885-015-1438-z)
Supplement: Additional file 1: Table S1. — Primer sequences of genes. Table S2. Oligonucleotide sequences for transfection. Table S3. Western Blot primary antibodies. [file 12885_2015_1438_MOESM1_ESM.doc]

**Supplementary Table S1. Primer sequences of genes**

| Gene name | Primer sequences |
| --- | --- |
| miR-217 | Forward: 5’-TACTGCATCAGGAACTGATTGGA-3’ |
| U6 | Forward: 5’-CTCGCTTCGGCAGCACA-3’ |
| AEG-1 | Forward: 5’- TTGAAGTGGCTGAGGGTGAA-3’  Reverse: 5’- TACGCTGCTGTCGTTTCTCT-3’ |
| GAPDH | Forward: 5’-CCCCGGTTTCTATAAATTGAGC-3’  Reverse: 5’-CACCTTCCCCATGGTGTCT-3’ |

**Supplementary Table S2. Oligonucleotide sequences for transfection**

| Gene name | Sequences | |
| --- | --- | --- |
| miR-217 | | 5’-UACUGCAUCAGGAACUGAUUGGA-3’ |
| Mimics NC | | 5’-UUUGUACUACACAAAAGUACUG |
| miR-217 inhibitor | | 5’-AUGACGUAGUCCUUGACUAACCU-3’ |
| Inhibitor NC  AEG-1 siRNA | | 5’-AAACAUGAUGUGUUUUCAUGAC  5’ -GCTGACTGATTCTGGTTCAT-3’ |

**Supplementary Table S3. Western Blot primary antibodies.**

| Antibody | Corporation | Dilution Ratio |
| --- | --- | --- |
| Anti-GAPDH (#2118)  Anti-AEG-1 (13860-1-AP)  Anti-MMP2 (#4022)  Anti-MMP9 (#13667)  Anti-Bcl-2 (#2870)  Anti-Bax (#5023)  Anti-cyclinD1 (#2978) | Cell Signal Technology  Proteintech  Cell Signal Technology  Cell Signal Technology  Cell Signal Technology  Cell Signal Technology  Cell Signal Technology | 1:1000  1:500  1:1000  1:1000  1:500  1:500  1:1000 |

**Supplementary Figure legends**

**Supplementary Figure S1. The effect of AEG-1 expression level on survival of CRC patients.** Kaplan-Meier survival curve for CRC patients with AEG-1-high (n = 26) and AEG-1-low (n = 24) character. P value was obtained by a log-rank test.

**Supplementary Figure S2. Knockdown of AEG-1 inhibit malignant biological behavior in SW480 and SW620 cell lines.** **(A)** AEG-1 expression was downregulated after treated with siRNA-AEG-1 determined by qRT-PCR (left) and Western blot analysis (right). **(B)** Inhibition of AEG-1 expression repressed cell proliferation of SW480 and SW620 cells. **(C)** Silencing of AEG-1 led to repression of colony formation. **(D)** Knockdown of MAP4K4 weakened the ability of cell invasion. **(E)** Cell cycle was examined by flow cytometry. Silencing of MAP4K4 in SW480 and SW620 cells led to G0/G1 arrest. **(F)** The percentage of apoptotic cells increased through downregulation of AEG-1 in SW480 and SW620 cell lines. *P<0.05, **P<0.01

**Supplementary Figure S3. Rescue of miR-217 ectopic expression effects by simultaneous overexpression of AEG-1.** **(A)** Cell proliferation detected in SW620 cells at 1, 2, 3, 4 and 5 days after transfection. **(B)** Results of SW620 cell invasion across an 8-μm pore size membrane with Matrigel. **(C)** Cell cycle determined in SW620 cells 48 h after transfection by Propidium-iodide staining flow cytometry. **(D)** Cell apoptosis detected by Annexin-V/propidium iodide combined labeling flow cytometry in SW620 cells 48 h after transfection. *P<0.05, **P<0.01
