# Supplementary figures and images for: MicroRNA-217 functions as a prognosis predictor and inhibits colorectal cancer cell proliferation and invasion via an AEG-1 dependent mechanism
Source: BMC Cancer. 2015 May 28;15:437. doi: 10.1186/s12885-015-1438-z (PMC4446846; doi:10.1186/s12885-015-1438-z)

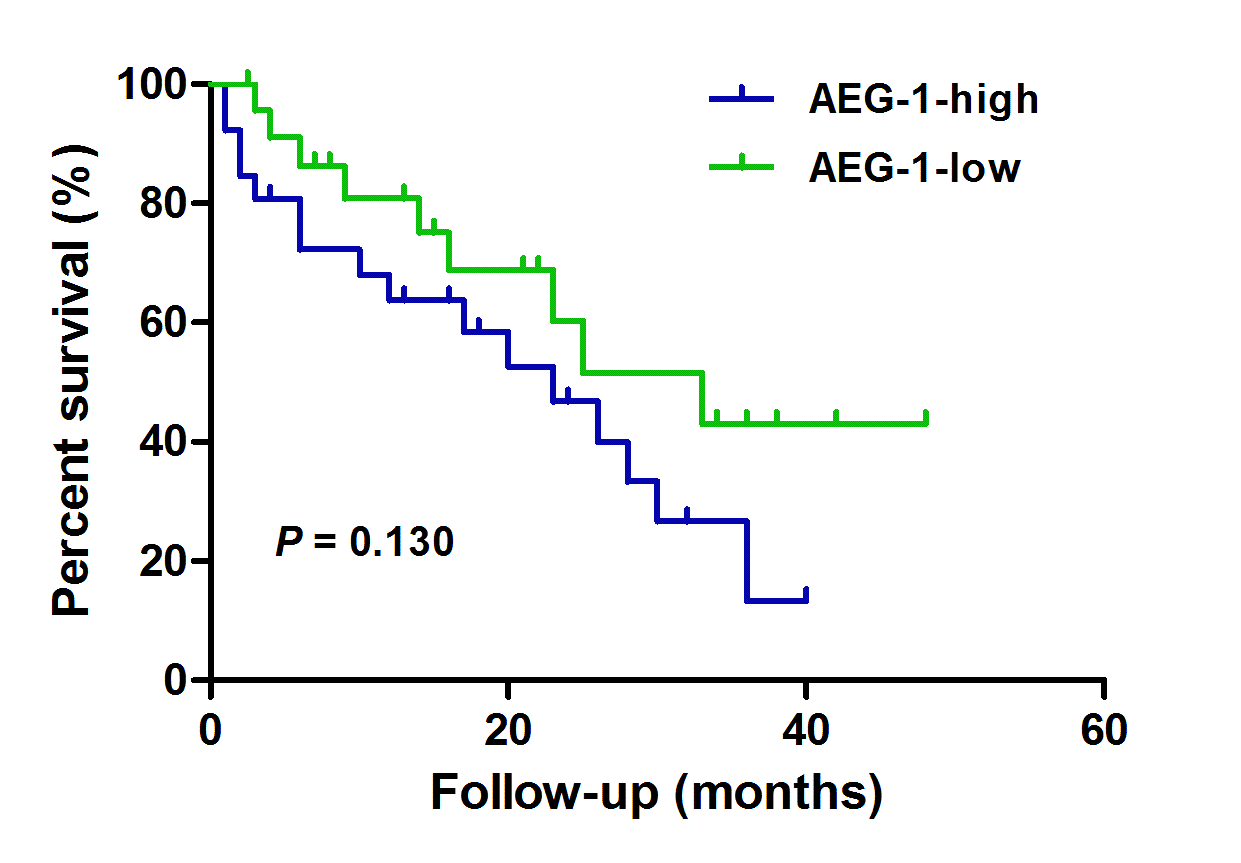

Supplement: Additional file 2: Figure S1. — The effect of AEG-1 expression level on survival of CRC patients. Kaplan-Meier survival curve for CRC patients with AEG-1-high (n = 26) and AEG-1-low (n = 24) character. P value was obtained by a log-rank test. [file 12885_2015_1438_MOESM2_ESM.tiff]

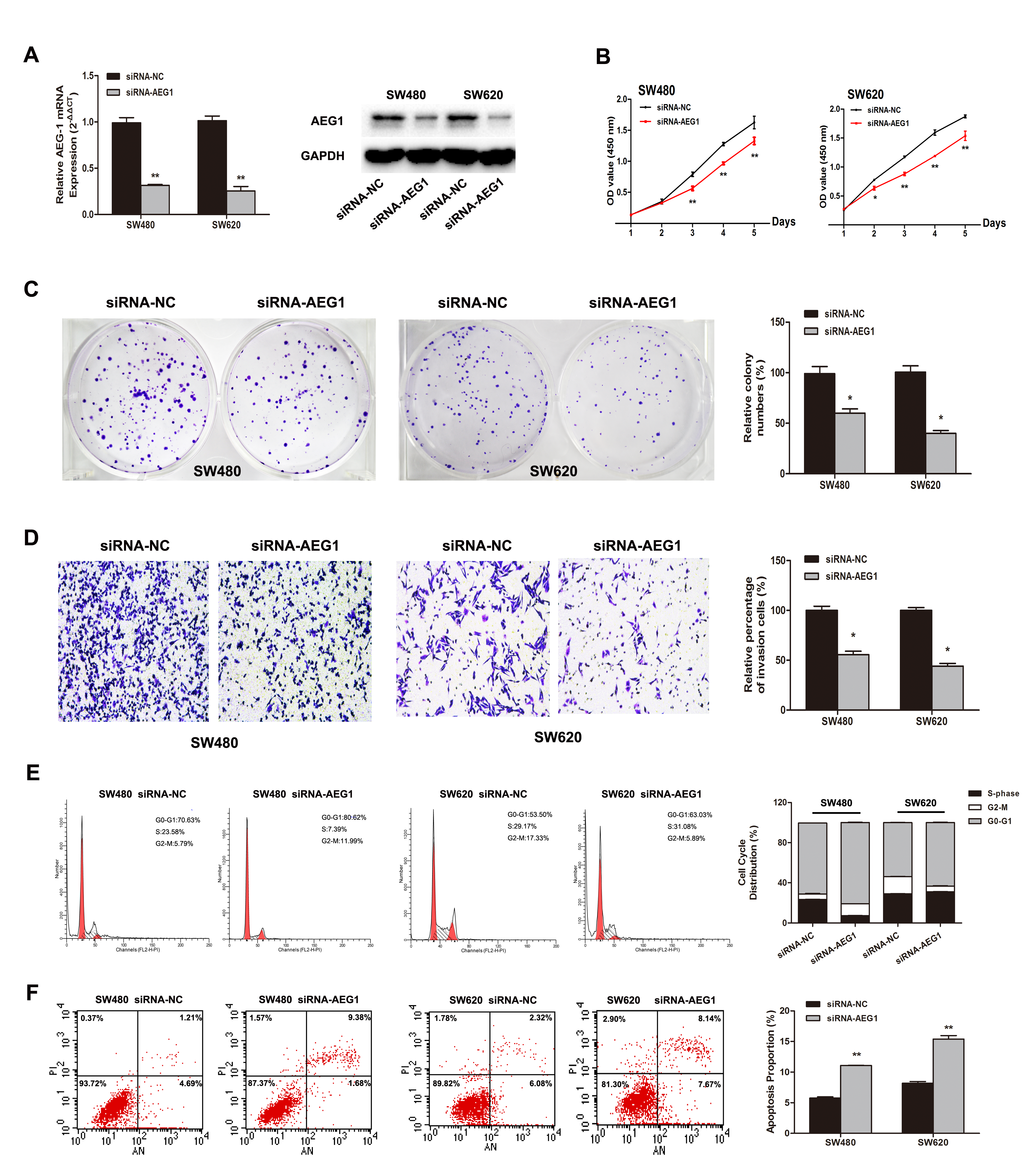

Supplement: Additional file 3: Figure S2. — Knockdown of AEG-1 inhibit malignant biological behavior in SW480 and SW620 cell lines. (A) AEG-1 expression was downregulated after treated with siRNA-AEG-1 determined by qRT-PCR (left) and Western blot analysis (right). (B) Inhibition of AEG-1 expression repressed cell proliferation of SW480 and SW620 cells. (C) Silencing of AEG-1 led to repression of colony formation. (D) Knockdown of MAP4K4 weakened the ability of cell invasion. (E) Cell cycle was examined by flow cytometry. Silencing of MAP4K4 in SW480 and SW620 cells led to G0/G1 arrest. (F) The percentage of apoptotic cells increased through downregulation of AEG-1 in SW480 and SW620 cell lines. *P < 0.05, **P < 0.01. [file 12885_2015_1438_MOESM3_ESM.tiff]

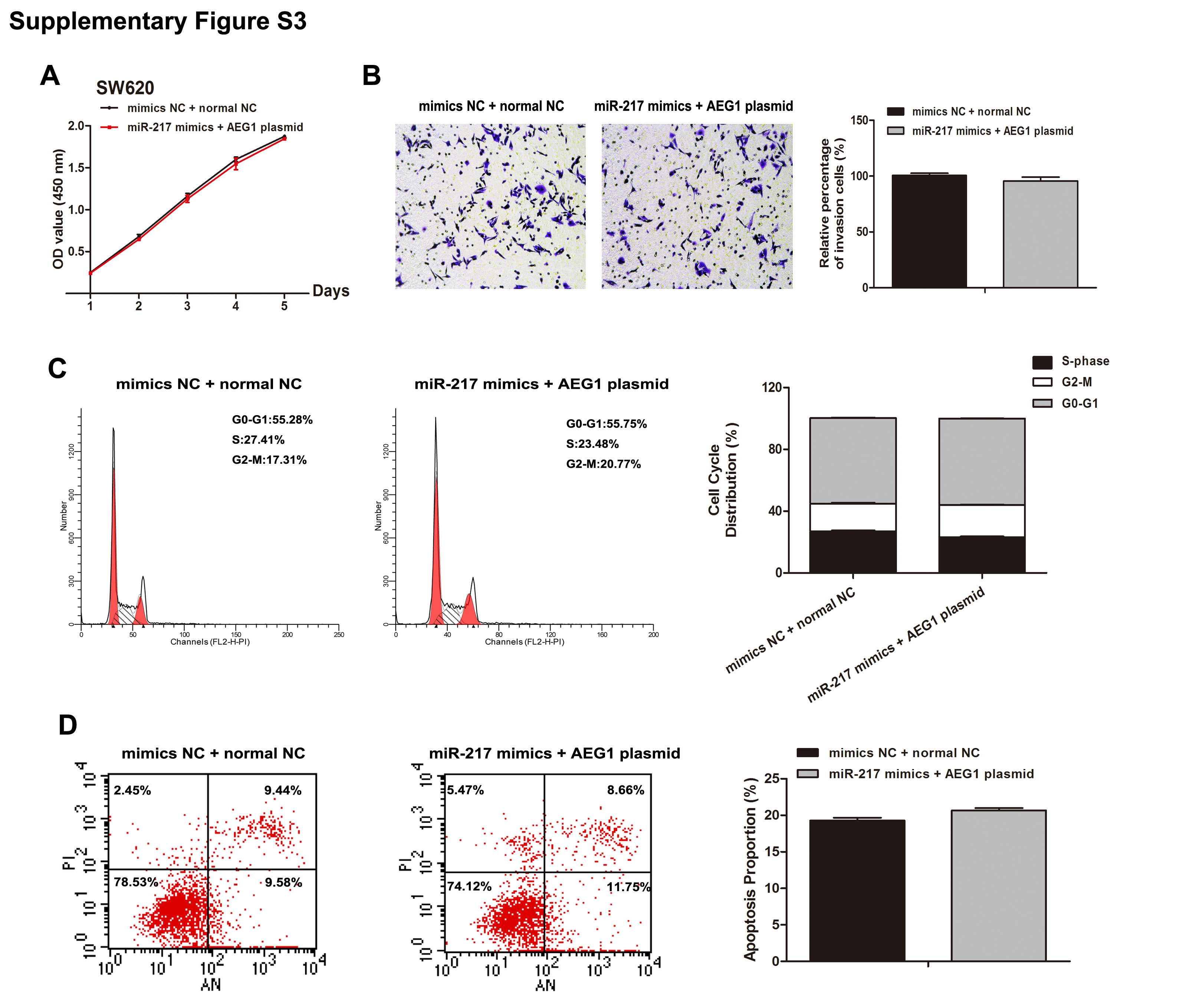

Supplement: Additional file 4: Figure S3. — Rescue of miR-217 ectopic expression effects by simultaneous overexpression of AEG-1. (A) Cell proliferation detected in SW620 cells at 1, 2, 3, 4 and 5 days after transfection. (B) Results of SW620 cell invasion across an 8-μm pore size membrane with Matrigel. (C) Cell cycle determined in SW620 cells 48 h after transfection by Propidium-iodide staining flow cytometry. (D) Cell apoptosis detected by Annexin-V/propidium iodide combined labeling flow cytometry in SW620 cells 48 h after transfection. *P < 0.05, **P < 0.01. [file 12885_2015_1438_MOESM4_ESM.tiff]
